# Integrated Care for Pregnant Women and Parents With Methamphetamine-Related Mental Disorders

**DOI:** 10.3389/fpsyt.2021.762041

**Published:** 2021-10-25

**Authors:** Johannes Petzold, Maik Spreer, Maria Krüger, Cathrin Sauer, Tobias Kirchner, Susanna Hahn, Ulrich S. Zimmermann, Maximilian Pilhatsch

**Affiliations:** ^1^Department of Psychiatry and Psychotherapy, Carl Gustav Carus University Hospital, Technische Universität Dresden, Dresden, Germany; ^2^Department of Psychiatry and Biobehavioral Sciences, University of California, Los Angeles, Los Angeles, CA, United States; ^3^Department of Addiction Medicine and Psychotherapy, kbo-Isar-Amper-Klinikum München-Ost, Haar, Germany; ^4^Department of Psychiatry and Psychotherapy, Elblandklinikum Radebeul, Radebeul, Germany

**Keywords:** methamphetamine use disorder, drug dependence, addiction, ADHD, depression, pregnancy, multimodal therapy, outcome prediction

## Abstract

**Background:** Methamphetamine use is a rapidly increasing cause of morbidity and mortality. Pregnant women and new parents who consume methamphetamine are at high risk since they seldom seek health services despite having multiple needs. We addressed this care gap by implementing an easily accessible program that pools resources from psychiatric, obstetric, and pediatric departments as well as community and government agencies.

**Method:** This real-life observational study evaluated an integrated care program in 27 expecting parents and 57 parents of minors. The outcome criteria were treatment retention, psychosocial functioning, and abstinence. We compared participant demographics according to outcome and applied ordinal logistic regression to predict treatment success.

**Results:** Patients received integrated care for almost 7 months on average. Nearly half achieved stable abstinence and functional recovery. Only one pregnant woman dropped out before a care plan could be implemented, and all women who gave birth during treatment completed it successfully. Three-fourths of patients had psychiatric comorbidities. Patients with depressive disorders were almost 5 times less likely to succeed with treatment. Attention-deficit hyperactivity disorder (ADHD) was diagnosed in nearly 30% of patients who dropped out of a care plan, which was about 4 times more often than in the successful outcome group.

**Conclusion:** Our program engaged pregnant women and parents in treatment and helped them recover from methamphetamine-related mental disorders. Management of comorbid ADHD and depression should be an integral part of care initiatives to counter the methamphetamine crisis that affects parents and children across the globe.

## Introduction

Methamphetamine use continues to rise on a global scale (1), and methamphetamine-involved overdoses claim lives in staggering numbers (2). This humanitarian crisis is further fueled by maternal, fetal, and child deaths related to methamphetamine exposure (3–6). Moreover, ample evidence implicates methamphetamine in lasting health and psychosocial problems that severely affect parents and their children (3–5). Women who use methamphetamine are burdened by mental disorders in the perinatal period (5), and parents who use methamphetamine are overstrained by their caregiving roles in often adverse living conditions (7). Infants can experience serious damage, such as microcephaly, due to intrauterine methamphetamine exposure (5, 8). Child development is compromised as a result of these factors, as evidenced by lower IQ scores as well as internalizing (e.g., depression) and externalizing symptoms (e.g., aggression) (5, 7, 8). Despite the urgency for prevention and intervention, the uptake of antenatal, pediatric, and mental health services is low and too late in this population (5, 9, 10). Moreover, pregnant women who use methamphetamine leave substance use treatment against professional advice even more often than pregnant women who use other illicit drugs or alcohol (11).

The stark contrast between care needs and utilization highlights the importance of developing programs that are easily accessible and appealing to those impacted by methamphetamine. Pregnancy and parenthood can create a strong motivation for abstinence (7), but treatment may not be accessed due to fear of stigma and punitive measures (5, 12, 13). Thus, transparency and the commitment to keeping families together are paramount. To reach women of childbearing age, expecting parents, and parents of minors, different avenues within the healthcare system, social services, and the community should be used. Linking these avenues also promises to help families achieve lasting health benefits as continuity of care is instrumental in sustaining abstinence (14, 15).

We are aware of only one study that at least partly addressed this care gap. This study compared methamphetamine-specific psychoeducation with a program also covering relationship and parenting skills tailored to South-African pregnant women (12). Both group interventions reduced methamphetamine use and risky sexual behavior, and 92% of participants completed the 4-session comprising interventions (12). Only 14% had been in substance use treatment before, indicating that access to care can be improved substantially (12).

Acknowledging the unmet needs of families affected by substance use disorders, the concept “Mama denk an mich” (MAMADAM, “Mommy think of me”) was developed to deliver coordinated care across disciplines and settings. After having demonstrated the feasibility of MAMADAM (9, 16), we here present its potential to improve the mental health of pregnant women and parents who use methamphetamine. The findings on outcome prediction provide critical information for patient assessment and program optimization. With this report, we hope to encourage the implementation and study of similar initiatives to promote the well-being of parents and children impacted by methamphetamine.

## Method

This is a real-life observational study of integrated care for expecting and new parents with methamphetamine-related psychiatric disorders, which was approved by the ethics committee at the Carl Gustav Carus Faculty of Medicine at the Technische Universität Dresden, Germany.

### Care Model

MAMADAM is a family-centered concept that pools resources from psychiatric, obstetric, and pediatric departments as well as local drug counseling and child welfare services. The concept and its elements draw on the available evidence and best practice for the management of methamphetamine-related disorders (8). Services provided through MAMADAM are easily accessible and flexible, ranging from health information for women of childbearing age to comprehensive support for families across care sectors. Shared decision-making and the participation of patients in multidisciplinary meetings ensure that treatment is matched to their preferences and needs. Other engagement strategies are calling patients who missed appointments and offering provider continuity whenever feasible. The following outlines the parts that are directed to the mental health of expecting and new parents. The coordination and specifics of MAMADAM, including information on obstetric and pediatric services, are described elsewhere (9, 16).

All expecting and new parents who use methamphetamine and present to our psychiatric department are considered for enrollment in MAMADAM. Access is facilitated by referrals from healthcare providers and by psychiatric consultations at the obstetric and pediatric departments. Motivational interviewing is used to develop personalized care plans after assessing mental illness and methamphetamine-related medical sequelae. Patients are seen by psychiatrists and psychotherapists on an outpatient basis from several times a week to once a month as needed. Inpatient and day treatments are provided whenever necessary.

An individual session introduces a methamphetamine-specific relapse prevention program that combines aspects of psychoeducation, motivational interviewing, and cognitive behavior therapy (17, 18). Psychotherapists deliver this program in 15 sessions of 50 min to a maximum of 5 patients. Individual psychotherapy and other group therapies are also available (e.g., exercise classes, social skills training, psychoeducation for major depression).

Social workers and occupational therapists help patients enhance their functioning and well-being in areas such as work, housing, and childcare. They partner with local and government agencies to furnish services ranging from community connections to intensive home support. Home care includes random drug screening, but most patients are called into the clinic once in 6 days on average. Urine is collected under direct observation followed by temperature measurement to minimize manipulation.

### Analytic Strategy

We studied a naturalistic sample of patients with methamphetamine-related disorders who received psychiatric care within MAMADAM since its start in 2016 and left or completed treatment before September 13, 2019. Outcome was classified as early dropout (before implementation of a care plan), partial completion of the program (late dropout), and successful completion. Successful completion was defined as a mutually agreed program discharge, which required continuous abstinence, stable housing, financial security, psychosocial functioning, and a support system. Psychosocial functioning required patients to perform daily activities in ways that were gratifying to them while meeting the demands of their dependents (e.g., safe environment, loving relationship) and the community (e.g., engagement in employment). Support usually involved primary care physicians, private psychiatrists, drug counseling centers, and child welfare services.

We used SPSS 27 (IBM, Armonk, NY, USA) and a significance level of 0.05 (two-tailed) for all analyses. Statistics were performed on complete data from all patients unless stated otherwise. We compared participant demographics and the duration of psychiatric care within MAMADAM according to outcome, using Pearson's chi-square-test, Fisher's exact-test, and Bonferroni-adjusted pairwise comparisons for categorical variables. Histograms, normal quantile-quantile plots, and normality tests (Kolmogorov-Smirnov, Shapiro-Wilk) determined the tests for continuous variables (one-way independent ANOVA, Kruskal-Wallis, Mann-Whitney). To identify predictors of outcome, we built a base model with all participant demographics that had complete data and met the assumptions of ordinal logistic regression. We then progressively removed non-significant variables to produce a parsimonious model. Variables that differed considerably between outcome groups and significant predictors were tested for associations, reporting the phi or Spearman's coefficient. These associations were not corrected for multiple comparisons.

## Results

We studied 84 patients with methamphetamine-related mental disorders (1 × F15.0, 8 × F15.1, 74 × F15.2, 1 × F19.2; diagnosed according to the International Classification of Diseases, 10th revision). This sample comprised 27 expecting parents and 57 parents of minors (mean age of child ± SD, min-max: 19.02 ± 30.59, 1–144 months; newborns counted as 1 month). Sixteen patients (19.0%) dropped out before receiving a care plan, 27 (32.1%) completed part of the program, and 41 (48.8%) transitioned successfully to community care. Average program participation was over 6 months (mean ± SD, min-max: 202.49 ± 167.42, 0–793 days) and not statistically different between patients who partially and those who successfully completed treatment (*n* = 68, *U* = 655.00, *z* = 1.27, *p* = 0.203).

Table 1 displays demographics according to outcome, showing no statistical differences in sex, age, years of methamphetamine use, and prior addiction rehab. The proportion of pregnant women was significantly lower in the early than in the late dropout group, and all women who gave birth during treatment completed it successfully. Three-fourths of patients had psychiatric comorbidities. Groups were comparably affected except for attention-deficit hyperactivity disorder (ADHD). Although there was only a trend to an overall significant difference between groups, ADHD was significantly less common in patients successfully than in those partially completing the program.

**Table 1:** Participant demographics.

	**Early dropout** **(*n* = 16)**	**Partial completion** **(*n* = 27)**	**Successful** **completion (*n* = 41)**	**Group differences**
**Sex**				${\text{X}}_{\left(2\right)}^{2}$ = 0.921, *p* = 0.696^F^
Women	13 (81.3)	23 (85.2)	37 (90.2)	
Men	3 (18.8)	4 (14.8)	4 (9.8)	
**Age**	31.63 ± 5.25 (21–38)	28.89 ± 5.02 (18–38)	28.51 ± 6.19 (18–41)	*F*_(2,81)_ = 1.809, *p* = 0.170^A^
**Expecting parents^#^**	1 (6.3)_a_	12 (44.4)_b_	14 (34.1)_a, b_	${\text{X}}_{\left(2\right)}^{2}$ = 6.867, *p* = 0.032^C^^*^
Pregnant women	1 (7.7)_a_	11 (47.8)_b_	14 (37.8)_a, b_	*n* = 73 women, ${\text{X}}_{\left(2\right)}^{2}$ = 5.995, *p* = 0.048^F^^*^
Becoming a parent during treatment				
	0 (0.0)_a, b_	0 (0.0)_b_	6 (42.9)_a_	*n* = 27 expecting parents^#^, ${\text{X}}_{\left(2\right)}^{2}$ = 7.163, *p* = 0.023^F^^*^
	0 (0.0)_a, b_	0 (0.0)_b_	6 (42.9)_a_	*n* = 26 pregnant women, ${\text{X}}_{\left(2\right)}^{2}$ = 6.686, *p* = 0.026^F^^*^
**Years of regular methamphetamine use**	6.43 ± 6.27 (0–23)	7.62 ± 6.34 (0–24)	6.11 ± 5.92 (0–25)	*n* = 76, *H*_(2)_ = 1.122, *p* = 0.571^K^
**Prior addiction rehab**	9 (56.3)	13 (48.1)	15 (36.6)	${\text{X}}_{\left(2\right)}^{2}$ = 2.077, *p* = 0.354^C^
**Current psychiatric comorbidity**				
Due to substance use (2 × F10.1, 8 × F10.2, 1 × F11.2, 3 × F12.1, 35 × F12.2, 4 × F19.2)	6 (37.5)	14 (51.9)	26 (63.4)	${\text{X}}_{\left(2\right)}^{2}$ = 3.256, *p* = 0.196^C^
Depressive disorder (1 × F32.0, 3 × F32.1, 1 × F33, 1 × F33.4, 1 × F33.8)	2 (12.5)	4 (14.8)	1 (2.4)	${\text{X}}_{\left(2\right)}^{2}$ = 3.713, *p* = 0.151^F^
Personality disorder (2 × F60.30, 7 × F60.31, 3 × F60.8)	4 (25.0)	3 (11.1)	5 (12.2)	${\text{X}}_{\left(2\right)}^{2}$ = 1.869, *p* = 0.465^F^
Attention-deficit hyperactivity disorder (F90.0)	3 (18.8)_a, b_	8 (29.6)_b_	3 (7.3)_a_	${\text{X}}_{\left(2\right)}^{2}$ = 5.897, *p* = 0.059^F^
Any (the above plus 1 × F40.1, 2 × F43.2, 1 × F55.2, 1 × F63, 1 × F63.0, 2 × F63.8, 3 × F70.0, 1 × F70.8, 1 × F91.1, 1 × F91.3, 1 × Q86.0)	11 (68.8)	20 (74.1)	32 (78.0)	${\text{X}}_{\left(2\right)}^{2}$ = 0.549, *p* = 0.757^F^
Any except due to substance use	6 (37.5)	14 (51.9)	14 (34.1)	${\text{X}}_{\left(2\right)}^{2}$ = 2.191, *p* = 0.334^C^

*Mental illness was diagnosed according to the International Classification of Diseases, 10th revision. Any psychiatric comorbidity comprised social phobias, adjustment disorders, abuse of laxatives, habit and impulse disorders, mild intellectual disability, conduct disorders, and fetal alcohol syndrome. Statistics are based on complete data from the entire sample (N = 84) unless stated otherwise. Data are number of patients (percentage within outcome category) or group mean ± SD (min-max). Percentages with the same subscript do not significantly differ from each other (Bonferroni adjusted)*.

*A, one-way independent ANOVA; C, Pearson's chi-square test; F, Fisher's exact-test; K, Kruskal-Wallis test*.

# *Twenty-six pregnant women + one expecting father*.

* *Statistically significant*.

When accounting for the order of outcomes (worst to best: early dropout, partial completion, successful completion), depression, substance use comorbidity, and prior addiction rehab emerged as significant predictors. Patients with substance use comorbidities were more likely to have a better outcome, whereas the opposite applied to patients with depressive disorders and patients with prior addiction rehab. Table 2 lists the unique (net) contribution of each significant predictor to variations in outcomes (controlled for the other significant predictors).

**Table 2:** Ordinal logistic regression model.

	**Parameter**	***B* (SE)**	**OR**	***p***
Threshold	Early dropout to partial completion	−0.16 (0.77)		
	Partial to successful completion	1.50 (0.79)		
Prior addiction rehab	No (vs. yes)	0.92 (0.45)	2.52	0.039^*^
Current substance use comorbidity		−1.14 (0.45)	0.32	0.012^*^
Current depressive disorder		1.58 (0.78)	4.87	0.041^*^

*This model was built from a base model by progressively removing the non-significant variables (first to last: current personality disorder, sex, expecting parents, current attention-deficit hyperactivity disorder, age). N = 84 with complete data on all variables. ${\text{X}}_{\left(3\right)}^{2}$ = 11.452, p = 0.010, Nagelkerke pseudo R^2^ = 14.6%*.

* *Statistically significant*.

None of the outcome predictors or variables with considerable group differences were significantly related to one another (see Table 3). Yet, there was one trend-level significant association with 59.3% of expecting parents compared with 36.8% of parents having prior addiction rehab. Of note, expecting parents, patients with prior addiction rehab, and patients with ADHD featured a significantly longer use of methamphetamine. Regular methamphetamine use was also numerically longer for patients who dropped out compared with those who completed MAMADAM successfully.

**Table 3:** Associations between correlates of outcome.

	**Expecting parents**	**Prior addiction rehab**	**Current substance use comorbidity**	**Current depressive disorder**	**Current attention-deficit hyperactivity disorder**	**Years of regular methamphetamine use**
Expecting parents		*r*_ϕ_ = 0.211 *p* = 0.053^C^	*r*_ϕ_ = 0.165 *p* = 0.131^C^	*r*_ϕ_ = −0.208 *p* = 0.091^F^	*r*_ϕ_ = 0.034 *p* = 1.000^F^	*r*_S_ = 0.258 *p* = 0.024^*^
Prior addiction rehab			*r*_ϕ_ = 0.180 *p* = 0.099^C^	*r*_ϕ_ = −0.007 *p* = 1.000^F^	*r*_ϕ_ = 0.054 *p* = 0.623^C^	*r*_S_ = 0.372 *p* = 0.001^*^
Current substance use comorbidity				*r*_ϕ_ = 0.101 *p* = 0.449^F^	*r*_ϕ_ = −0.043 *p* = 0.695^C^	*r*_S_ = 0.157 *p* = 0.175
Current depressive disorder					*r*_ϕ_ = 0.096 *p* = 0.595^F^	*r*_S_ = −0.066 *p* = 0.572
Current attention-deficit hyperactivity disorder						*r*_S_ = 0.285 *p* = 0.013^*^

*Categorical variables (expecting parents, prior addiction rehab, comorbidities) are coded as 0 = no and 1 = yes. Pearson's chi-square (C) and Fisher's exact-tests (F) are based on complete data from the entire sample (N = 84). Spearman's correlations (r_S_) are based on n = 76 due to missing data on years of methamphetamine use*.

* *Statistically significant*.

## Discussion

This study evaluated the real-world adherence to integrated care by pregnant women and parents with methamphetamine-related mental disorders. Despite being challenged by their roles and psychiatric comorbidities, nearly half of our patients completed treatment successfully, and only one pregnant woman dropped out before receiving a care plan. These data support the notion that pregnancy and parenthood can be motivators for abstinence (7, 8, 19). We are not aware of any study that evaluated a comparable concept for this population, but methamphetamine use is generally associated with high dropout rates (8, 19). Less than one-fourth of patients with methamphetamine as their primary drug completed 180 days of outpatient drug treatment, a duration previously identified as necessary for treatment success (20). Moreover, pregnant women using methamphetamine left substance use treatment against professional advice even more often than those using other illicit drugs or alcohol (11).

Three-fourths of our patients had at least one other mental disorder, which reflects the high psychiatric morbidity reported in association with methamphetamine use (8, 19). Patients with depressive disorders were almost 5 times more likely to have less treatment success than patients without such a diagnosis. This aligns with an outpatient treatment study for methamphetamine dependence in which higher baseline depression predicted methamphetamine use before discharge and poorer treatment attendance (21). Although not predicted by baseline depression scores, methamphetamine use 3 years post-treatment was associated with major depression at follow-up (21). Together, these findings emphasize the need for routine assessment and treatment of depression. Exercise and psychological interventions can reduce depressive symptoms, but methamphetamine-induced cognitive deficits complicate the latter (8, 19). Medications including antidepressants have largely failed and their potential for drug interaction effects has been highlighted (8, 19). Yet, recent research suggests that glutamatergic modulators with antidepressant and pro-cognitive properties may prove effective in treating methamphetamine use disorder and comorbid depression (22).

ADHD was diagnosed in almost one-third of patients who dropped out of an established care plan. This proportion was about 4 times as high as in the successful outcome group. Moreover, ADHD was associated with more years of regular methamphetamine use, which was also numerically longer for patients who dropped out. Routine assessment for ADHD seems therefore imperative, not least because some patients report using methamphetamine to treat ADHD symptoms (8). The risk of misuse and drug interactions warrants close monitoring of pharmacotherapy, with long-acting formulations recommended if stimulant medication is prescribed (19).

A diagnosis of substance use comorbidity predicted a better outcome, which indicates that the benefits of MAMADAM extend to other drugs. Experiencing a substance use comorbidity might increase readiness for change and treatment. Patients with prior addiction rehab were, in comparison, more likely to have a worse outcome. Similarly, previous drug treatment predicted treatment attrition in pregnant women using methamphetamine weekly or more (11). Prior treatment could indicate greater disease severity or longer disease duration, as patients with prior addiction rehab featured more years of regular methamphetamine use. Of note, more years of regular methamphetamine use predicted poor outcome in a study of the methamphetamine-specific group psychotherapy that we provide in MAMADAM (18). The data collectively highlight that patients with previous drug treatment require close monitoring and greater support. Recognizing the value of their treatment experiences may be a way to better meet their needs and expectations, which should improve retention in care and health outcomes.

### Strengths and Limitations

To our knowledge, this is the first work evaluating the adherence to integrated care by pregnant women and parents with methamphetamine-related mental disorders. We did not include a control condition but the exploratory study of a naturalistic sample in the real world can provide outcome predictors of direct importance for patient care. Of note, we report significant predictors from a parsimonious regression model that did not control for non-significant variables and only considered variables with complete data. ADHD was not among the significant predictors in our regression model yet more prevalent in patients who dropped out, with the late dropout group statistically differing from the successful outcome group. We believe that ADHD and other comorbidities, such as depressive and personality disorders, are underreported for patients dropping out of MAMADAM early since these diagnoses require thorough assessments in drug-free intervals. Lastly, we did not collect follow-up data, but treatment success required stable abstinence and continuing care, which are critical factors for long-term recovery.

## Conclusion

Integrated care is a promising strategy for pregnant women and parents with methamphetamine-related mental disorders. Pregnancy and parenthood provide opportunities to motivate change and engage a population that hardly accesses treatment despite the high psychiatric morbidity. Comorbid ADHD and depression warrant close monitoring as they jeopardize treatment engagement and success. Given the little information on the management of these conditions in the context of methamphetamine use, research is imperative to provide evidence-based interventions. Moreover, integrated care concepts should be disseminated to counter the increasing methamphetamine crisis that affects parents and children across the globe.

## Data Availability Statement

The raw data supporting the conclusions of this article will be made available by the authors, without undue reservation.

## Ethics Statement

The studies involving human participants were reviewed and approved by the Ethics Committee at the Carl Gustav Carus Faculty of Medicine at the Technische Universität Dresden, Germany. Written informed consent for participation was not required for this study in accordance with the national legislation and the institutional requirements.

## Author Contributions

UZ, MP, MS, and SH contributed to the development of the care concept. MS, SH, TK, UZ, and MP were members of the care team. MP, UZ, CS, and MK designed the study. MP and JP obtained funding. MK, CS, MS, MP, and TK collected the data. JP analyzed and interpreted the data and wrote the manuscript. All authors contributed to manuscript revision, as well as read and approved the final version.

## Funding

This work was supported by the German Research Foundation [TRR 265 (23)]. The article processing charge was paid from the Publication Fund of the Technische Universität Dresden. The funders were not involved in the conceptualization of the study, the collection, analysis, interpretation of data, the preparation of the manuscript, or the decision to submit it for publication.

## Conflict of Interest

The authors declare that the research was conducted in the absence of any commercial or financial relationships that could be construed as a potential conflict of interest.

## Publisher's Note

All claims expressed in this article are solely those of the authors and do not necessarily represent those of their affiliated organizations, or those of the publisher, the editors and the reviewers. Any product that may be evaluated in this article, or claim that may be made by its manufacturer, is not guaranteed or endorsed by the publisher.

## Abbreviations

ADHD: attention-deficit hyperactivity disorder
MAMADAM: “Mama denk an mich”, “Mommy think of me”.

## References

[1] UNODC. World Drug Report 2020, Booklet 2 Drug Use and Health Consequences. United Nations publication, Vienna (2020).

[2] Kariisa M Scholl L Wilson N Seth P Hoots B. Drug overdose deaths involving cocaine and psychostimulants with abuse potential - United States, 2003-2017. Morb Mortal Wkly Rep. (2019) 68:388–95. 10.15585/mmwr.mm6817a3 31048676 PMC6541315

[3] Admon LK Bart G Kozhimannil KB Richardson CR Dalton VK Winkelman TNA. Amphetamine- and opioid-affected births: incidence, outcomes, and costs, United States, 2004-2015. Am J Public Health. (2019) 109:148–54. 10.2105/AJPH.2018.304771 30496001 PMC6301406

[4] Dinger J Hinner P Reichert J Rüdiger M. Methamphetamine consumption during pregnancy - effects on child health. Pharmacopsychiatry. (2017) 50:107–13. 10.1055/s-0042-122711 28178739

[5] Perez FA Blythe S Wouldes T McNamara K Black KI Oei JL. Prenatal Methamphetamine- impact on the mother and child - a review. Addiction. (2021). 10.1111/add.15509. [Epub ahead of print]. 33830539

[6] Tse R Kesha K Morrow P Glenn C Stables S. Commentary on: Kenneally M, Byard RW. Increasing methamphetamine detection in cases of early childhood fatalities. J Forensic Sci. (2020) 65:1384. 10.1111/1556-4029.14459 32510633

[7] Dyba J Moesgen D Klein M Leyendecker B. Methamphetamine use in German families: parental substance use, parent-child interaction and risks for children involved. Subst Use Misuse. (2019) 54:583–91. 10.1080/10826084.2018.1528459 30636479

[8] Braunwarth W-D Christ M Dirks H Dyba J Härtel-Petri R Harfst T. S3 *Practice Guideline Methamphetamine-Related Disorders*. 1st ed. Berlin: Agency for Quality in Medicine (2016).

[9] Dinger J Näther N Wimberger P Zimmermann US Schmitt J Reichert J. Increasing consumption of crystal meth in saxony and its risks for mother and child - experiences at a level I perinatal center from a pediatric viewpoint. Z Geburtshilfe Neonatol. (2017) 221:73–80. 10.1055/s-0043-102953 28561211

[10] Stevens S Rogers J Dansereau L DellaGrotta S Lester BM Wouldes TA. Prospective study of service use in the year after birth by women at high risk for antenatal substance use and mental health disorders. Int J Ment Health Addict. (2019) 19:1005–1018. 10.1007/s11469-019-00207-w

[11] Lindsay B Albrecht J Terplan M. Against professional advice: treatment attrition among pregnant methamphetamine users. Subst Abuse Rehabil. (2011) 2:189–95. 10.2147/SAR.S25083 24474856 PMC3846313

[12] Jones HE Myers B O'Grady KE Gebhardt S Theron GB Wechsberg WM. Initial feasibility and acceptability of a comprehensive intervention for methamphetamine-using pregnant women in South Africa. Psychiatry J. (2014) 2014:929767. 10.1155/2014/929767 24829904 PMC3994903

[13] Lewis D. We were wrong about “crack babies”: are we repeating our mistake with “meth babies”? MedGenMed. (2005) 7:30. 16614652 PMC1681748

[14] Chi FW Parthasarathy S Mertens JR Weisner CM. Continuing care and long-term substance use outcomes in managed care: early evidence for a primary care-based model. Psychiatr Serv. (2011) 62:1194–200. 10.1176/ps.62.10.pss6210_1194 21969646 PMC3242696

[15] Manning V Garfield JB Best D Berends L Room R Mugavin J. Substance use outcomes following treatment: findings from the Australian Patient Pathways Study. Aust N Z J Psychiatry. (2017) 51:177–89. 10.1177/0004867415625815 26769978

[16] Groß C Hahn S Spreer M Behrendt S Dinger J Reichert J. “Mama denk' an mich” (MAMADAM) - ein multimodales Therapieprogramm für suchtkranke Schwangere, Mütter und Väter im Rahmen der psychiatrischen Institutsambulanz. SUCHT. (2018) 64:97–108. 10.1024/0939-5911/a000533

[17] Groß C Schützwohl M Mayer-Pelinski R Hasler H Kirchner T Scheck A. “CrystalClean” - a German-language manual for qualified detoxification and motivation treatment in cases of “crystal meth” dependency - feasibility and acceptance. Psychiatr Prax. (2020) 47:22–8. 10.1055/a-1003-5148 31910457

[18] Petzold J Weber B Bassett TR Bauer M Bernhardt N Gross C. Effectiveness of the first German-language group psychotherapy manual to accompany short-term treatment in methamphetamine dependence. Front Psychiatry. (2020) 11:130. 10.3389/fpsyt.2020.00130 32180742 PMC7059436

[19] Grigg J Manning V Arunogiri S Volpe I Frei M Phan V. Methamphetamine Treatment Guidelines: Practice Guidelines for Health Professionals. 2nd ed. Richmond, VA, Victoria: Turning Point. (2018).

[20] Maglione M Chao B Anglin MD. Correlates of outpatient drug treatment drop-out among methamphetamine users. J Psychoactive Drugs. (2000) 32:221–8. 10.1080/02791072.2000.10400232 10908011

[21] Glasner-Edwards S Marinelli-Casey P Hillhouse M Ang A Mooney LJ Rawson R. Depression among methamphetamine users: association with outcomes from the methamphetamine treatment project at 3-year follow-up. J Nerv Ment Dis. (2009) 197:225–31. 10.1097/NMD.0b013e31819db6fe 19363377 PMC2749575

[22] Petzold J Szumlinski KK London ED. Targeting mGlu(5) for methamphetamine use disorder. Pharmacol Ther. (2021) 224:107831. 10.1016/j.pharmthera.2021.107831 33705840

[23] Heinz A Kiefer F Smolka MN Endrass T Beste C Beck A. Addiction research consortium: losing and regaining control over drug intake (ReCoDe)-From trajectories to mechanisms and interventions. Addict Biol. (2020) 25:e12866. 10.1111/adb.12866 31859437

